# The Role of Sleep Quality and Physical Activity Level on Gait Speed and Brain Hemodynamics Changes in Young Adults—A Dual-Task Study

**DOI:** 10.3390/ejihpe12110117

**Published:** 2022-11-17

**Authors:** Marina Saraiva, Maria António Castro, João Paulo Vilas-Boas

**Affiliations:** 1 RoboCorp Laboratory, i2A, Polytechnic Institute of Coimbra, 3046-854 Coimbra, Portugal; 2 Faculty of Sports and CIAFEL, University of Porto, 4200-450 Porto, Portugal; 3 Department of Mechanical Engineering, CEMMPRE, University of Coimbra, 3030-788 Coimbra, Portugal; 4 Sector of Physiotherapy, School of Health Sciences, Polytechnic Institute of Leiria, 2411-901 Leiria, Portugal; 5 LABIOMEP-UP, Faculty of Sports and CIFI2D, University of Porto, 4200-450 Porto, Portugal

**Keywords:** simultaneous tasks, physical activity, cognitive, speed gait, sleep, fNIR, prefrontal cortex

## Abstract

Walking requires attentional resources, and the studies using neuroimage techniques have grown to understand the interaction between cortical activity and motor performance. Previous studies reported a decline in gait performance and changes in the prefrontal cortex (PFC) activity during a dual-task performance compared to walking only. Some lifestyle factors, such as sleep and physical activity (PA) levels, can compromise walking performance and brain activity. Nonetheless, the studies are scarce. This study aimed to assess gait speed and hemodynamic response in the PFC during a cognitive dual-task (cog-DT) compared to walking only, and to analyze the correlation between PA and sleep quality (SQ) with gait performance and hemodynamic response in the PFC during a single task (ST) and cog-DT performance in young adults. A total of 18 healthy young adults (mean age ± SD = 24.11 ± 4.11 years) participated in this study. They performed a single motor task (mot-ST)—normal walking—and a cog-DT—walking while performing a cognitive task on a smartphone. Gait speed was collected using a motion capture system coupled with two force plates. The hemoglobin differences (Hb-diff), oxyhemoglobin ([oxy-Hb]) and deoxyhemoglobin ([deoxy-Hb]) concentrations in the PFC were obtained using functional near-infrared spectroscopy. The SQ and PA were assessed through the Pittsburg Sleep Quality Index and International Physical Activity Questionnaire-Short Form questionnaires, respectively. The results show a decrease in gait speed (*p* < 0.05), a decrease in [deoxy-Hb] (*p* < 0.05), and an increase in Hb-diff (*p* < 0.05) and [oxy-Hb] (*p* > 0.05) in the prefrontal cortex during the cog-DT compared to the single task. A positive correlation between SQ and Hb-diff during the cog-DT performance was found. In conclusion, the PFC’s hemodynamic response during the cog-DT suggests that young adults prioritize cognitive tasks over motor performance. SQ only correlates with the Hb-diff during the cog-DT, showing that poor sleep quality was associated with increased Hb-diff in the PFC. The gait performance and hemodynamic response do not correlate with physical activity level.

## 1. Introduction

It is common to perform two tasks simultaneously in everyday life, such as walking while playing a game on a smartphone, walking while talking to other people, or maintaining a standing posture while reading a newspaper. The capacity to perform two tasks concurrently is called the dual-task paradigm [1]. However, generally, when people perform two tasks simultaneously, the attention is divided between both tasks, which can result in a decline in the performance of one or both tasks due to the limited ability to share attentional resources [2,3,4].

Previous studies reported that walking requires attentional resources and is not just an automated motor activity [2,5,6]. Thus, studies that assessed gait performance when simultaneously performing cognitive or motor secondary tasks showed a decline in gait performance in dual-task conditions. For example, a reduction in gait speed during walking when performing a secondary task compared to normal walking was observed in young adults [7,8,9], older adults [10,11], and in people with neurological diseases [12,13].

The prefrontal cortex plays an essential role in executive functions and gait control [14,15]. Furthermore, neuroimaging studies have grown to understand brain activity resulting from the interaction between motor and cognitive task performance. Some neuroimaging techniques used to assess the cognitive resources and brain regions involved in walking performance under dual-task conditions are functional magnetic resonance imaging (fMRI) [16], positron-emission tomography (PET) [17], electroencephalography (EEG) [18], and functional near-infrared spectroscopy (fNIRS) [19]. fNIR has advantages over others because it is portable and can be used during motion and in natural environments [19,20]. In addition, it measures the changes in oxy and deoxyhemoglobin concentrations associated with neural activity [21,22]. A systematic review showed that increased brain activity could lead to more walking impairments due to the use of more attentional resources during a dual-task, especially in older adults with neurological diseases [23]. Another study using fNIR concluded that walking while performing a cognitive task increased brain activation in prefrontal regions and decreased gait performance compared to normal walking in healthy young adults [7].

The attentional demands during walking while performing a secondary task [24] can depend on the task’s type and complexity and the subjects’ age [6]. However, some lifestyles can compromise walking performance and brain activity, such as sleep quality and physical activity; nevertheless, studies are scarce. Existing studies suggest that the oxyhemoglobin was higher after a whole night’s sleep than at the beginning of the night [25]. Others reported that sleep plays an important role in the processes of learning and memory [26], that physical activity benefits the executive function [27] and gait speed [28], and that sedentarism [29], poor sleep quality [30], and more than 8 h of sleep duration [31] can be associated with lower gait speed.

Gait speed is considered the sixth “vital sign” because it is easily measurable and provides essential information about the functional status [32,33]. Moreover, gait speed can be correlated with cognitive impairment and the risk of falls [34,35]. For that reason, we consider it important to investigate gait speed and neural activity during dual-task conditions in young adults to obtain more knowledge about the interaction between motor and cognitive task performance, and its relationship with sleep quality and physical activity, to detect early signs of impairments. Therefore, this study aims to assess the changes in gait speed and hemodynamics response on the prefrontal cortex resulting from the addition of a cognitive task during walking (cognitive dual-task) compared to normal walking (single task). Furthermore, we also intend to determine the correlation between sleep quality and physical activity level with gait performance and brain hemodynamics changes during the dual-task. We hypothesized that: (i) the young adults would demonstrate a reduction in gait speed and an increase in hemodynamics response in the prefrontal cortex during the performance of a dual-task compared to a single task; (ii) sleep quality and physical activity level would correlate with gait performance and brain hemodynamics changes during a dual-task in young adults.

## 2. Materials and Methods

### 2.1. Participants

A total of 18 healthy young adults, aged between 18 and 35 years, voluntarily participated in this study and signed the informed consent form (sample characteristics are in Table 1). They reported having no known history of cardiovascular, musculoskeletal, neurological, vestibular, or cognitive disorders or of taking medications. The Ethics Committee of the Polytechnic Institute of Coimbra approved the study (27_CEPC2/2019).

### 2.2. Procedure

We considered it important to use tasks similar to everyday life; therefore, as the smartphone is a massively used electronic device that can modify gait behavior [36], in this study, in the dual-task condition, the participants performed the cognitive task using a smartphone while walking. Based on previous studies [19,37,38,39,40], the task protocol used in this research is the following:

Single motor task (mot-ST)—The participants were instructed to walk at a self-selected preferred walking speed and regularly pace back and forth along an 8 m walkway for 60 s.

Single cognitive task (cog-ST)—The participants performed a cognitive task on a smartphone based on working memory tasks [2] and verbalized their responses while sitting on a chair for 60 s.

Cognitive dual-task (cog-DT)—The participants were instructed to walk while simultaneously performing a cognitive task on a smartphone for 60 s.

The gait performance (gait speed) and hemodynamics changes in the prefrontal cortex were collected during the single task and cognitive dual-task. The cognitive task performance was measured through the percentage of correct answers collected during cognitive single- and dual-task conditions. The motor task performance was determined through the collection of gait speed and hemodynamics changes in the prefrontal cortex during normal walking and the cognitive dual-task. Each task was performed for 60 s, twice with 45 s rest. The young adults were not given any instructions regarding which task to prioritize during the cog-DT and performed the tasks randomly to minimize the learning factor. To maintain ecological validity, the participants performed the cognitive single- and dual-task with their usual smartphone and held it with their preferred hand or both hands.

### 2.3. Instruments and Data Analysis

Gait speed data were collected with ten Oquos^®^ Optoelectronic cameras of high speed and a resolution of 1.3 to 12 megapixels, with a 200 Hz measurement frequency, coupled with two force plates (Bertec Corporation, Columbus, OH 43229, USA; AMTI, Watertown, MA, USA) using an optical motion capture system (Qualysis AB, Göteborg, Sweden) and the Qualisys Track Manager v2.15 software (Qualisys AB, Götebor, Sweden). According to Wilken et al. [41], 53 reflective markers on defined anatomical landmarks were placed by one experienced researcher. Furthermore, marker clusters were placed on the thighs and shanks to improve segment tracking quality. Gait speed data were filtered with a 6-Hz Butterworth low-pass filter and processed using the Visual 3D software (C-Motion, Germantown, MD, USA).

The hemodynamic changes in the prefrontal cortex were recorded using fNIR100A-2 (Biopac System Inc., Goleta, CA, USA) equipment attached to the forehead. This fNIR device has 16 recording channels with a source–detector separation of 2.5 cm and records at a frequency of 2 Hz, detecting infrared light wavelengths at 730 nm and 850 nm. Cognitive Optical Brain Imaging (COBI) was used for data acquisition, and the fNIRSoft professional software for data processing (Biopac software). After a visual inspection to remove low-quality channels, the raw signals were filtered using a low-pass finite impulse response (FIR) filter, with an order of 20 Hamming, and a cutoff frequency of 0.1 Hz [42,43,44] to eliminate confounding physiological noise. Next, the motion artifacts were removed using a sliding-window motion artifact rejection (SMAR) algorithm [42]. The changes in oxyhemoglobin ([oxy-Hb]) and deoxyhemoglobin ([deoxy-Hb]] concentrations relative to a 10 s baseline were recorded according to the modified Beer–Lambert Law [44]. The hemoglobin difference (Hb-diff = [oxy-Hb] − [deoxy-Hb]) was also extracted for the assessment of hemodynamics response in the prefrontal cortex.

Sleep quality was assessed using the Pittsburg Sleep Quality Index (PSQI). This self-report questionnaire assesses sleep quality over the previous month. A global score above 5 indicates poor sleep quality. The global sleep quality score ranges from 0 to 21 [45]. The Portuguese version of the PSQI presents adequate validity and reliability (Cronbach’s α of 0.70) for assessing sleep quality [46] such as other PSQI versions [47,48].

Physical activity level was assessed using a self-report questionnaire, the International Physical Activity Questionnaire-Short Form (IPAQ-SF). It assesses the intensity of physical activity in MET-min/week over the last seven days [49]. The physical activity score was obtained according to the IPAQ instrument protocol [50].

### 2.4. Statistical Analysis

The descriptive variables, such as the sample’s socio-demographic characteristics, the IPAQ-SF and PSQI total scores, were presented as mean and ± SD (standard deviation), and the physical activity level was presented as frequencies. The Shapiro–Wilk test confirmed the non-normality of the data. The Wilcoxon signed-rank test was used to compare gait speed, cognitive task performance, [oxy-Hb], [deoxy-Hb] and Hb-diff between single- and dual-task performance. The data were presented as the median and interquartile range (IQR).

The Spearman’s rho test was used to correlate sleep quality and physical activity level with gait performance and hemodynamics changes in the prefrontal cortex during motor single-task and cognitive dual-task conditions.

All analyses were performed using IBM-SPSS 25.0 software and the significance level was set at *p* < 0.05.

## 3. Results

### 3.1. Gait Speed and Hemodynamic Changes in the Prefrontal Cortex

The difference in walking performance and hemodynamics changes in the prefrontal cortex between normal walking and the cognitive dual-task are represented in Table 2.

When we added a cognitive task to the motor task of walking, a decrease in gait speed, a decrease in [deoxy-Hb], an increase in [oxy-Hb], and a higher Hb-diff in the prefrontal cortex were found compared to normal walking. Only in the oxyhemoglobin concentration were no differences found between normal walking and the cog-DT.

### 3.2. Cognitive Task Performance

There was an increase in cognitive task performance from the single cognitive task (cognitive task on a smartphone in a seated position) to the cognitive dual-task (walking while performing a cognitive task on a smartphone). The median percentage of correct responses increased from the single cognitive task (58.33 (42.13–69.91)%) to the cognitive dual-task (78.70 (64.35–96.30)%); this difference was significant (*p* < 0.001).

### 3.3. Relationship between Physical Activity and Sleep Quality with Gait Performance and Hemodynamics Response under Single- and Dual-Task Conditions

Young adults presented a total IPAQ-SF score of 3701.06 ± 3460.345 MET-min/week and a total PSQI score of 5.18 ± 3.28. Concerning the physical activity level, according to the IPAQ-SF, 41.2% have a high level of physical activity, 41.2% have a moderate physical activity, and 17.6% have a low level of physical activity.

The analysis showed a moderate, positive, and significant correlation between the sleep quality and the Hb-diff during cog-DT performance. However, there were no significant relationships between the other outcomes analyzed (*p* > 0.05) (see Table 3).

## 4. Discussion

This study investigated the influence of cognitive task on gait speed performance and the hemodynamics response in the prefrontal cortex while walking (cognitive dual-task) compared to normal walking, and the association between physical activity level and sleep quality with gait performance and hemodynamics response in the prefrontal cortex under normal walking and cognitive dual-task conditions in young adults.

Our results show that, when a cognitive task is added to walking, a decline in gait speed and changes in prefrontal cortex activation are detected, suggesting that both tasks share neural networks [14,15] and that more attentional resources to perform the tasks are needed. Furthermore, the young adults showed that they allocated more attentional recourses to perform the cognitive task to the detriment of walking performance, because they demonstrated an improvement in the cognitive task performance from the cog-ST to the cog-DT, verified by the increase in the percentage of correct answers. The hemodynamics response increased in the prefrontal cortex from normal walking to the cognitive dual-task, showing an increase in Hb-diff and a decrease in deoxyhemoglobin concentration. Although the oxyhemoglobin concentration also increased from the mot-ST to the cog-DT, no differences were found between the two conditions, which may be related to the fact that the participants verbalized their answers during the cognitive task performance. The reduction in oxyhemoglobin can result from a decrease in cerebral blood flow and cerebral oxygenation as a consequence of hypocapnia caused by verbalization [51].

Similar to our research, a decrease in gait speed and higher prefrontal activation from normal walking to the cognitive dual-task was observed in other studies. For example, a study using fNIR showed that young adults, when performing a cognitive dual-task, decreased gait performance and increased activation in the prefrontal area than in normal walking [7]. In addition, a review paper reported that most studies showed increased prefrontal activity during dual-task performance compared to usual walking [52]. Another systematic review reported that gait control requires cognitive resources, and there is generally a decline in gait performance when there is simultaneous involvement of cognitive tasks while walking [24]. Concerning the tasks used in our study, a systematic review that analyzed the influence of tasks performed using a smartphone while walking showed a decline in gait performance in dual-task conditions in most studies assessed [38].

Regarding the correlation analysis between physical activity and sleep quality, only a correlation between sleep quality and the difference in hemoglobin during the cog-DT was found. This correlation suggests that poor sleep quality is associated with a greater difference in hemoglobin in prefrontal cortex activation in dual-task conditions. We suppose that this may be due to a compensatory mechanism resulting from a higher mental effort (amount of cognitive resources allocated to perform a task [53]) and consequent overload in the recruitment of cognitive resources, leading to lower system efficiency caused by poor sleep quality. In children, it appears that the worst dual-task performance is associated with disrupted sleep, a higher quantity of REM (rapid eye-movement) sleep related to lower gait variability, and a higher cognitive performance associated with a greater quantity of slow-wave sleep [54]. Another study showed that poor sleep quality was related with a slower normal walking speed in adults [31]. In addition, a decrease in gait speed and higher gait variability were associated with a lower sleep efficiency during a dual-task but not while performing a single task in older people [55].

Although no correlations were found in this study between physical activity and gait performance and hemodynamics response, some studies report that moderate-to-vigorous-intensity physical activity improves gait speed (over 50 years) [28]. In addition, another study reported that moderate-to-high levels of physical exercise positively affect executive functions in middle-aged and older individuals [27]. A study in young adults suggested that good sleep quality was associated with higher gait speed in single-task conditions, but did not investigate during dual-task conditions [30].

In this study, we used self-reported questionnaires to assess sleep quality and physical activity, which may have conditioned our results in the correlation tests. Thus, we considered this a limitation of this study together with the small sample size. Therefore, we recommended future studies that objectively measure these outcomes and correlate them with gait performance and brain activity during performing tasks. Furthermore, in the cognitive dual-task used in this study, most young adults reduced the swing movement of their arms, and their field of vision decreased due to manipulating the smartphone, which may have contributed to the decrease in gait speed. Therefore, in addition to the tasks used in this study, we also suggest performing cognitive tasks without a smartphone to better understand the influence of cognitive tasks on gait speed under dual-task conditions. Another limitation was that we did not monitor blood pressure or breathing cycle, considering that these parameters can influence fNIR measurements [20,56]. Future studies should consider these parameters.

Our study contributed to understanding the interaction between motor and cognitive performance under dual-task conditions and how activity in the prefrontal cortex changes. Moreover, it showed that sleep quality might interfere with hemodynamics response in the prefrontal cortex during dual-task conditions. In this way, implementing strategies to improve sleep quality can be helpful to the functioning of the prefrontal cortex. For example, moderate physical activity can be a tool to enhance sleep quality [57]. Furthermore, facing the changes in motor and cognitive performance from normal walking to the cognitive dual-task and the increased risk of injury due to smartphone use reported in other research [58,59], dual-task training can be used to reduce the interference between motor and cognitive performance, minimizing the risk of injuries or falls [60].

## 5. Conclusions

The hemodynamic response in the prefrontal cortex during a cog-DT suggests that young adults prioritize cognitive tasks over motor performance and allocate more attentional recourses in the cognitive task than gait performance. The gait speed decreases, the hemoglobin difference in the prefrontal cortex increases, and the deoxyhemoglobin concentration decreases during a cognitive dual-task compared to normal walking. Sleep quality only correlates with the Hb-diff during the cog-DT, showing that poor sleep quality was associated with increased Hb-diff in the prefrontal cortex. The gait performance and hemodynamics response do not correlate with the physical activity level. Future studies that objectively assess physical activity and sleep quality are recommended to investigate and clarify the correlation between these factors with gait speed performance and brain activity. Implementing clinical practices that improve sleep quality and motor and cognitive performance during dual-task conditions can help optimize the interaction between motor and cognitive systems.

## Figures and Tables

**Table 1:** Sample’s socio-demographic characteristics.

Variables	Sample *n* = 18
Age (years)	24.11 ± 4.11
Height (m)	1.74 ± 0.07
Body mass (Kg)	79.92 ± 14.24
Body Mass Index (Kg/m^2^)	26.36 ± 4.13

**Table 2:** Gait performance and hemodynamics changes in the prefrontal cortex between normal walking and cognitive dual-task conditions.

Outcomes	Single Motor Task	Cog-DT	*p*-Value ^1^
Gait speed (m/s)	1.05 (0.94–1.18)	0.95 (0.90–1.10)	0.006 *
[oxy-Hb] (μ mol/L)	0.18 (−0.41–0.73)	0.30 (−0.57–0.73)	0.501
[deoxy-Hb] (μ mol/L)	−1.09 (−1.27–(−0.31))	−1.41 (−2.02–(−0.79))	0.039 *
Hb-diff (μ mol/L)	0.92 (0.22–1.59)	1.27 (0.54–2.80)	0.039 *

[oxy-Hb], oxyhemoglobin concentration; [deoxy-Hb], deoxyhemoglobin concentration; Hb-diff, difference between oxy and deoxyhemoglobin concentrations; cog-DT, cognitive dual-task. * *p* < 0.05: Comparison between single motor task and cognitive dual-task (^1^ Wilcoxon signed-rank test).

**Table 3:** Relationship between total IPAQ-SF and PSQI scores with gait performance and hemodynamics response under single- and dual-task conditions.

Outcomes	IPAQ-SF Total Score		PSQI Total Score	
	Spearman’s Rho	*p*-Value	Spearman’s Rho	*p*-Value
cog-DT [oxy-Hb]	0.392	0.119	0.326	0.202
cog-DT [deoxy-Hb]	−0.100	0.701	−0.258	0.318
cog-DT Hb-diff	0.235	0.363	**0.522**	**0.032**
mot-ST [oxy-Hb]	0.109	0.688	0.002	0.996
mot-ST [deoxy-Hb]	0.156	0.564	−0.294	0.269
mot-ST Hb-diff	0.085	0.753	0.253	0.344
Gait speed: cog-DT	−0.243	0.348	0.301	0.241
Gait speed: mot-ST	−0.191	0.462	0.133	0.610

IPAQ-SF, International Physical Activity Questionnaire-Short Form; PSQI, Pittsburg Sleep Quality Index; [oxy-Hb], oxyhemoglobin concentration (μ mol/L); [deoxy-Hb], deoxyhemoglobin concentration (μ mol/L); Hb-diff, difference between oxy and deoxyhemoglobin concentrations (μ mol/L); cog-DT, cognitive dual-task; mot-ST, single motor task. Spearman’s correlation test. Bold values with *p* < 0.05.

## Data Availability

Not applicable.

## References

[1] Macpherson S.E. (2018). Definition: Dual-tasking and multitasking. Cortex A J. Devoted Study Nerv. Syst. Behav..

[2] Bayot M., Dujardin K., Tard C., Defebvre L., Bonnet C.T., Allart E., Delval A. (2018). The interaction between cognition and motor control: A theoretical framework for dual-task interference effects on posture, gait initiation, gait and turning. Neurophysiol. Clin..

[3] Huang H.J., Mercer V.S. (2001). Dual-Task Methodology: Applications in Studies of Cognitive and Motor Performance in Adults and Children. Pediatr. Phys. Ther..

[4] Plummer P., Eskes G. (2015). Measuring treatment effects on dual-task performance: A framework for research and clinical practice. Front. Hum. Neurosci..

[5] Yogev-Seligmann G., Hausdorff J.M., Giladi N. (2008). The Role of Executive Function and Attention in Gait. Mov. Disord..

[6] Woollacott M., Shumway-Cook A. (2002). Attention and the control of posture and gait: A review of an emerging area of research. Gait Posture.

[7] Mirelman A., Maidan I., Bernad-Elazari H., Nieuwhof F., Reelick M., Giladi N., Hausdorff J.M. (2014). Increased frontal brain activation during walking while dual tasking: An fNIRS study in healthy young adults. J. Neuroeng. Rehabil..

[8] Schabrun S.M., Hoorn W.V.D., Moorcroft A., Greenland C., Hodges P. (2014). Texting and Walking: Strategies for Postural Control and Implications for Safety. PLoS ONE.

[9] Beurskens R., Steinberg F., Antoniewicz F., Wolff W., Granacher U. (2016). Neural Correlates of Dual-Task Walking: Effects of Cognitive versus Motor Interference in Young Adults. Neural Plast..

[10] Beurskens R., Bock O. (2013). Does the walking task matter? Influence of different walking conditions on dual-task performances in young and older persons. Hum. Mov. Sci..

[11] Júnior R.C.F., Porto J.M., Marques N.R., Magnani P.E., de Abreu D.C.C. (2017). The effects of a simultaneous cognitive or motor task on the kinematics of walking in older fallers and non-fallers. Hum. Mov. Sci..

[12] Liu Y.-C., Yang Y.-R., Tsai Y.-A., Wang R.-Y., Lu C.-F. (2018). Brain Activation and Gait Alteration During Cognitive and Motor Dual Task Walking in Spectroscopy Study. IEEE Trans. Neural Syst. Rehabil. Eng..

[13] Hunter S.W., Divine A., Frengopoulos C., Montero-Odasso M. (2018). A framework for secondary cognitive and motor tasks in dual-task gait testing in people with mild cognitive impairment. BMC Geriatr..

[14] Fuster J.M. (2001). The Prefrontal Cortex—An Update: Time is of the Essence. Neuron.

[15] Suzuki M., Miyai I., Ono T., Oda I., Konishi I., Kochiyama T., Kubota K. (2004). Prefrontal and premotor cortices are involved in adapting walking and running speed on the treadmill: An optical imaging study. Neuroimage.

[16] Bürki C.N., Bridenbaugh S.A., Reinhardt J., Stippich C., Kressig R.W., Blatow M. (2017). Imaging gait analysis: An fMRI dual task study. Wiley Brain Behav..

[17] Szturm T., Kolesar T.A., Mahana B., Goertzen A.L., Hobson D.E., Marotta J.J., Strafella A.P., Ko J.H. (2021). Changes in Metabolic Activity and Gait Function by Dual-Task Cognitive Game-Based Treadmill System in Parkinson’s Disease: Protocol of a Randomized Controlled Trial. Front. Aging Neurosci..

[18] Possti D., Fahoum F., Sosnik R., Giladi N., Hausdorff J.M., Mirelman A., Maidan I. (2021). Changes in the EEG spectral power during dual-task walking with aging and Parkinson’s disease: Initial findings using Event-Related Spectral Perturbation analysis. J. Neurol..

[19] Herold F., Wiegel P., Scholkmann F., Thiers A., Hamacher D., Schega L. (2017). Functional near-infrared spectroscopy in movement science: A systematic review on cortical activity in postural and walking tasks. Neurophotonics.

[20] Scholkmann F., Kleiser S., Metz A.J., Zimmermann R., Pavia J.M., Wolf U., Wolf M. (2014). A review on continuous wave functional near-infrared spectroscopy and imaging instrumentation and methodology. Neuroimage.

[21] Villringer A., Chance B. (1997). Non-invasive optical spectroscopy and imaging of human brain function. Trends Neurosci..

[22] Quaresima V., Ferrari M. (2019). Functional Near-Infrared Spectroscopy (fNIRS) for Assessing Cerebral Cortex Function During Human Behavior in Natural/Social Situations: A Concise Review. Organ. Res. Methods.

[23] Bishnoi A., Holtzer R., Hernandez M. (2021). Brain Activation Changes While Walking in Adults with and without Neurological Disease: Systematic Review Spectroscopy Studies. Brain Sci..

[24] Al-Yahya E., Dawes H., Smith L., Dennis A., Howells K., Cockburn J. (2011). Cognitive motor interference while walking: A systematic review and meta-analysis. Neurosci. Biobehav. Rev..

[25] Oniz A., Inanc G., Taslica S., Guducu C., Ozgoren M. (2019). Sleep is a Refreshing Process: An fNIRS Study. Front. Hum. Neurosci..

[26] Gudberg C., Johansen-Berg H. (2015). Sleep and Motor Learning: Implications for Physical Rehabilitation After Stroke. Front. Neurol..

[27] Berchicci M., Lucci G., Di Russo F. (2013). Benefits of physical exercise on the aging brain: The role of the prefrontal cortex. J. Gerontol. A Biol. Sci. Med. Sci..

[28] McMullan I.I., Bunting B.P., Mcdonough S.M., Tully M.A., Casson K. (2020). The association between light intensity physical activity with gait speed in older adults (≥50 years). A longitudinal analysis using the English Longitudinal Study of Ageing (ELSA). Aging Clin. Exp. Res..

[29] Willey J.Z., Moon Y.P., Kulick E.R., Cheung Y.K., Wright C.B., Sacco R.L., Elkind M.S.V. (2017). Physical inactivity predicts slow gait speed in an elderly multi-ethnic cohort study: The Northern Manhattan Study (NOMAS). Neuroepidemiology.

[30] Kasović M., Štefan A., Štefan L. (2021). The Associations Between Objectively Measured Gait Speed and Subjective Sleep Quality in First-Year University Students, According to Gender. Nat. Sci. Sleep.

[31] Wang L., Zou B. (2022). The Association Between Gait Speed and Sleep Problems Among Chinese Adults Aged 50 and Greater. Front. Neurosci..

[32] Fritz S., Lusardi M. (2009). White Paper: ‘Walking Speed: The Sixth Vital Sign. J. Geriatr. Phys. Ther..

[33] Montero-Odasso M., Schapira M., Soriano E.R., Varela M., Kaplan R., Camera L.A., Mayorga L.M. (2005). Gait Velocity as a Single Predictor of Adverse Events in Healthy Seniors Aged 75 Years and Older. J. Gerontol..

[34] Dyer A.H., Lawlor B., Kennelly S.P., for the NILVAD Study Group (2020). Gait speed, cognition and falls in people living with mild-to-moderate Alzheimer disease: Data from NILVAD. BMC Geriatr..

[35] Peel N.M., Alapatt L.J., Jones L.V., Hubbard R.E. (2019). The Association Between Gait Speed and Cognitive Status in Community-Dwelling Older People: A Systematic Review and Meta-analysis. J. Gerontol. A Biol. Sci. Med. Sci..

[36] Bovonsunthonchai S., Ariyaudomkit R., Susilo T.E., Sangiamwong P., Puchaphan P., Chandee S., Richards J. (2020). The impact of different mobile phone tasks on gait behaviour in healthy young adults. J. Transp. Health.

[37] Krasovsky T., Lanir J., Felberbaum Y., Kizony R. (2021). Mobile Phone Use during Gait: The Role of Perceived Prioritization and Executive Control. Int. J. Environ. Res. Public Health.

[38] Tan Y., Sun Y., Lang C., Wen Y. (2022). The Impact of Using Mobile Phones on Gait Characteristics: A Narrative Review. Appl. Sci..

[39] Crowley P., Madeleine P., Vuillerme N. (2016). Effects of Mobile Phone Use during Walking: A Review. Crit. Rev. Phys. Rehabil. Med..

[40] Takeuchi N., Mori T., Suzukamo Y., Tanaka N., Izumi S.-I. (2016). Parallel processing of cognitive and physical demands in left and right prefrontal cortices during smartphone use while walking. BMC Neurosci..

[41] Wilken J.M., Rodriguez K.M., Brawner M., Darter B.J. (2012). Reliability and minimal detectible change values for gait kinematics and kinetics in healthy adults. Gait Posture.

[42] Ayaz H., Izzetoglu M., Shewokis P.A., Onaral B. Sliding-window motion artifact rejection for Functional Near-Infrared Spectroscopy. Proceedings of the 2010 Annual International Conference of the IEEE Engineering in Medicine and Biology.

[43] Izzetoglu M., Chitrapu P., Bunce S., Onaral B. (2010). Motion artifact cancellation in NIR spectroscopy using discrete Kalman filtering. Biomed. Eng. Online.

[44] Herold F., Wiegel P., Scholkmann F., Müller N.G. (2018). Applications of Functional Near-Infrared Spectroscopy (fNIRS) Neuroimaging in Exercise–Cognition Science: A Systematic, Methodology-Focused Review. J. Clin. Med..

[45] Buysse D.J., Reynolds C.F., Monk T.H., Berman S.R., Kupfer D.J. (1998). The Pittsburgh Sleep Quality Index: A new instrument for psychiatric practice and research. Psychiatry Res..

[46] João K.A.D.R., Becker N.B., Jesus S.D.N., Martins R.I.S. (2017). Validation of the Portuguese version of the Pittsburgh Sleep Quality Index (PSQI-PT). Psychiatry Res..

[47] Backhaus J., Junghanns K., Broocks A., Riemann D., Hohagen F. (2002). Test-retest reliability and validity of the Pittsburgh Sleep Quality Index in primary insomnia. J. Psychosom. Res..

[48] Bertolazi A.N., Fagondes S.C., Hoff L.S., Dartora E.G., Miozzo I.C.D.S., de Barba M.E.F., Barreto S.S.M. (2011). Validation of the Brazilian Portuguese version of the Pittsburgh Sleep Quality Index. Sleep Med..

[49] Craig C.L., Marshall A.L., Sjöström M., Bauman A.E., Booth M.L., Ainsworth B.E., Pratt M., Ekelund U.L., Yngve A., Sallis J.F. (2003). International physical activity questionnaire: 12-Country reliability and validity. Med. Sci. Sports Exerc..

[50] IPAQ Group (2005). Guidelines for Data Processing Analysis of the International Physical Activity Questionnaire (IPAQ)-Short and Long Forms. http://www.ipaq.ki.se.

[51] Scholkmann F., Gerber U., Wolf M., Wolf U. (2013). End-tidal CO_2_: An important parameter for a correct interpretation in functional brain studies using speech tasks. Neuroimage.

[52] Vitorio R., Stuart S., Rochester L., Alcock L., Pantall A. (2017). Neuroscience and Biobehavioral Reviews fNIRS response during walking—Artefact or cortical activity ? A systematic review. Neurosci. Biobehav. Rev..

[53] Paas F.G.W.C., Van Merrienboer J.J.G. (1994). Variability of Worked Examples and Transfer of Geometrical Problem-Solving Skills: A Cognitive-Load Approach. J. Educ. Psychol..

[54] Möhring W., Urfer-Maurer N., Brand S., Holsboer-Trachsler E., Weber P., Grob A., Lemola S. (2019). The association between sleep and dual-task performance in preterm and full-term children: An exploratory study. Sleep Med..

[55] Agmon M., Shochat T., Kizony R. (2016). Sleep quality is associated with walking under dual-task, but not single-task performance. Gait Posture.

[56] Kirilina E., Jelzow A., Heine A., Niessing M., Wabnitz H., Brühl R., Ittermann B., Jacobs A.M., Tachtsidis I. (2012). The physiological origin of task-evoked systemic artefacts in functional near infrared spectroscopy. Neuroimage.

[57] Wang F., Boros S. (2021). The effect of physical activity on sleep quality: A systematic review. Eur. J. Physiother..

[58] Nasar J.L., Troyer D. (2013). Pedestrian injuries due to mobile phone use in public places. Accid. Anal. Prev..

[59] Haolan Z., Campbell I.M., Giang W.C. (2021). Phone-Related Distracted Walking Injuries as a Function of Age and Walking Environment. Proc. Hum. Factors Ergon. Soc. Annu. Meet..

[60] Pang M.Y.C., Yang L., Ouyang H., Lam F.M.H., Huang M., Jehu D.A. (2018). Dual-Task Exercise Reduces Cognitive-Motor Interference in Walking and Falls After Stroke. Stroke.

